# Hydrogen-Induced Ductility Loss of GH625 Superalloy Under Thermal Hydrogen Charging

**DOI:** 10.3390/ma18030526

**Published:** 2025-01-24

**Authors:** Jishun Zhang, Jiqing Zhao, Zhenyang Liao, Jia Yu, Rui Wang, Yongfu Sun, Gang Yang

**Affiliations:** 1 Research Institute for Special Steels, Central Iron and Steel Research Institute Co., Ltd. (CISRI), Beijing 100081, China; jishun_zhang@126.com (J.Z.); yanggang@nercast.com (G.Y.); 2 Hypervelocity Aerodynamics Institute, China Aerodynamics Researchand Development Center, Mianyang 621000, China; yujia_cardc@163.com; 3 Jiangyou Changcheng Special Steel Co., Ltd. of Pangang Group, Jiangyou 621704, China; wangruiman2024@163.com (R.W.); andysun126@126.com (Y.S.)

**Keywords:** hydrogen embrittlement, nickel-based superalloy, δ phase, hydrogen-induced cracking, intergranular fracture

## Abstract

The effect of thermal hydrogen charging on the tensile properties of GH625 superalloy was investigated. The results reveal that hydrogen significantly reduces the ductility of the GH625, leading to a shift from microvoid coalescence (MVC)-induced ductile fracture to intergranular (IG) brittle fracture. Random grain boundaries (GBs) are the primary sites for crack initiation. Hydrogen reduces the critical fracture stress of the δ phase at grain boundaries, causing cracking of the δ phase. Under the influence of hydrogen-enhanced localized plasticity (HELP) and hydrogen-enhanced decohesion (HEDE), the δ/γ interface debonds, forming microcracks that propagate along the fractured δ phase, leading to intergranular cracking. Annealing twin boundaries (TBs) serve as secondary sites for crack initiation. Hydrogen-induced local stress concentration promotes twin boundary sliding and hydrogen segregation reduces twin boundary cohesion strength, which is the primary cause of TB crack formation.

## 1. Introduction

GH625 is a solid-solution-strengthened superalloy with high strength, good weldability, and strong corrosion resistance, similar to Inconel 625 alloy [1]. It is widely used in hot-end components across aerospace, chemical, power, and other industries [2,3,4]. Due to its excellent performance, GH625 alloy is also considered for use in components exposed to hydrogen environments [5]. Most metals are susceptible to hydrogen embrittlement (HE) when exposed to hydrogen [6,7,8,9]. Face-centered cubic (FCC) metals are generally believed to have better resistance to HE due to their lower hydrogen diffusion rate and higher hydrogen solubility. However, nickel-based superalloys with FCC structure are susceptible to HE [10,11], limiting their use in hydrogen energy applications.

The HE of superalloys has been extensively investigated, yet the HE mechanisms remain incompletely understood. HEDE and HELP are two widely recognized mechanisms used to explain HE. The HEDE mechanism proposes that hydrogen weakens the cohesive strength of atomic bonds at phase interfaces and grain boundaries, resulting in crack initiation at lower stress levels [12,13]. Conversely, the HELP mechanism suggests that hydrogen reduces the critical stress required for dislocation movement during plastic deformation, significantly increasing dislocation mobility and resulting in premature material fracture [14,15]. Most research on hydrogen embrittlement of superalloys has focused on Inconel 718 [16,17,18,19,20,21,22,23,24], with relatively few studies addressing the HE mechanism of Inconel 625. Soundararajan et al. [25] found that the density of mobile dislocations in Inconel 625 increases with increasing hydrogen content, as determined using acoustic emission techniques. Feng et al. [26] studied the hydrogen embrittlement of arc additively manufactured 625 alloy and found that hydrogen accumulation at the γ/Laves phase interface weakened interfacial bonding strength and lowered the critical stress for crack propagation. Liu et al. [27] studied the effect of strain rate on hydrogen-induced ductility loss in Inconel 625 and reported an abnormal phenomenon: the alloy exhibited greater ductility loss at conventional strain rates compared to slow strain rates. They attributed this to a shift in deformation mode, from dislocation slip at slow strain rates to twinning at conventional strain rates. The aforementioned research does not fully explain the hydrogen embrittlement mechanism of GH625. Therefore, further investigation is needed to understand the hydrogen compatibility and embrittlement behavior of this alloy, providing guidance for the design of hydrogen embrittlement-resistant superalloys.

In this research, we evaluated the hydrogen embrittlement sensitivity of GH625 after thermal hydrogen charging using conventional tensile tests. The fracture and microstructural morphologies were analyzed in detail using scanning electron microscopy (SEM), while the microscopic deformation structures were examined using transmission electron microscopy (TEM) and electron channeling contrast imaging (ECCI). Based on these results, the hydrogen embrittlement failure mechanism of GH625 was discussed.

## 2. Experimental

### 2.1. Material

The experimental material used in this study was GH625 pipes with an outer diameter of 410 mm and an inner diameter of 265 mm, provided by Jiangyou Changcheng Special Steel Co., Ltd. (Mianyang, China). The alloy was produced using vacuum induction melting followed by vacuum arc remelting. After forging and piercing, the material underwent solution treatment at 935 °C for 2 h, followed by aging at 680 °C for 8 h. The chemical composition of the alloy is presented in Table 1.

### 2.2. Tensile Tests and Hydrogen Charging

The tensile test was performed at ambient temperature in air using a smooth round bar specimen with a diameter of 5 mm and a gauge length of 25 mm. The specimen was taken along the chord direction at half the thickness of the pipe. The strain rate for the tests was 2.5 × 10^−4^ s^−1^. The specimen was continuously charged with hydrogen in a gaseous environment at 20 MPa and 673 K for 336 h. Specimens with and without hydrogen charging are referred to as “H-charged” and “H-uncharged”, respectively. The hydrogen content of the specimens was determined to be approximately 32 ppm using an ONH3500 hydrogen determinator (Beijing, China).

Lu et al. studied the hydrogen permeation characteristics and reported the following equation for the hydrogen diffusion coefficient (*D*) in Inconel 625 [28]:

(1) $$D=1.82\times 10^{-7}\exp\left(-\frac{44.46\times 10^{3}}{RT}\right)\;m^{2}/s$$

where *R* represents the gas constant and *T* denotes the temperature. For a semi-infinite plate, the correlation between thickness (*Z*) and hydrogen homogenization time (*t*) is given by following equation [29]:

(2) $$Z≅1.12\sqrt{Dt}$$

According to the diffusion Equation (1), the diffusion coefficient of Inconel 625 is approximately 6.44 × 10^−11^ m^2^/s. Substituting this value into Equation (2), the hydrogen homogenization time in a 5 mm diameter tensile specimen was calculated to be 21 h. Therefore, it can be assumed that a homogeneous and saturated hydrogen distribution is achieved in the specimen after hydrogen charging.

### 2.3. Microstructure Characterization

**Figure 1:**
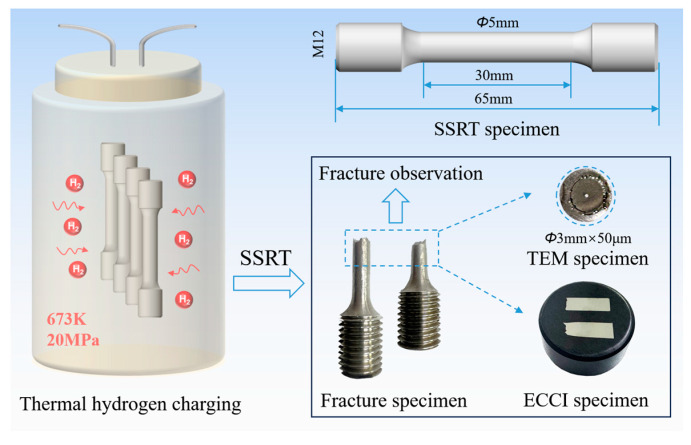
Experimental process and observation location.

The specimens were etched in a solution of 10 g CuCl_2_, 200 mL HCl, and 200 mL C₂H₅OH, and the microstructure was characterized using a JEOL JSM-7001F SEM (Tokyo, Japan). ECCI was used to characterize the microscopic deformation substructures near crack initiation and propagation sites. Prior to ECCI characterization, the specimens were vibratory polished to achieve a deformation-free surface. Furthermore, in addition, precipitates and micro-deformation structures were observed using a field emission TEM (JEOL JEM-F200, Tokyo, Japan). The TEM specimens were prepared by double-jet electropolishing using a solution of 10% HClO_4_ acid and 90% C_2_H_5_OH at −30 °C and 20 V. The experimental process and sample observation locations are illustrated in Figure 1.

## 3. Results

### 3.1. Initial Microstructure

**Figure 2:**
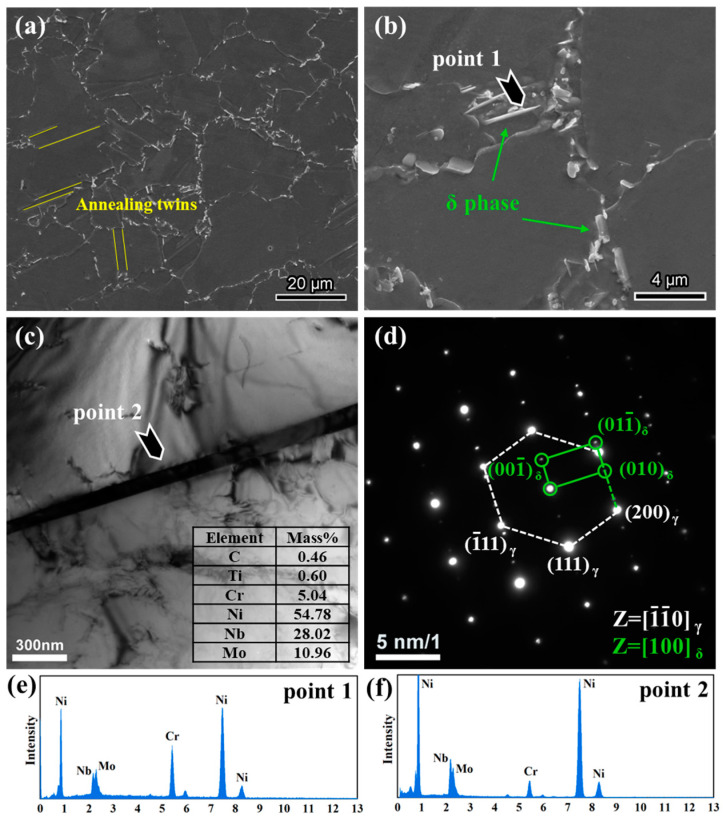
Microstructure of GH625 after heat treatment: (**a**,**b**) the SEM micrograph, TBs are marked by the yellow lines, δ phases are marked by the green arrows; (**c**) TEM BF image and the element contents of a needle-like δ phase; (**d**) SAED pattern of δ phase; (**e,f**) EDS results for point 1 and point 2.

Figure 2 displays the initial microstructure of GH625. The average grain size is approximately 22 μm, and the grains exhibit distinct annealing twins, some of which are highlighted with yellow lines. Additionally, numerous Nb-rich precipitates were observed, primarily in short rod-like or needle-like morphologies. These precipitates are mainly located at grain boundaries, with a smaller number present at twin boundaries and almost none within the grains. Selected area electron diffraction (SAED) analysis combined with energy-dispersive spectroscopy (EDS) confirmed that the Nb-rich phase is δ-Ni_3_Nb. Along the [110] zone axis of the matrix, the orientation relationship between the matrix and the δ-phase was found to be [110]_γ_∥[100]_δ_, (110)_γ_∥(100)_δ_. In Inconel 625, the δ phase typically forms between 750 °C and 980 °C [30]. To achieve higher strength, a relatively low solution temperature of 935 °C was used in this study, which may promote δ-phase precipitation. Additionally, due to the large size of the tube, the heating and cooling processes during forging and heat treatment are relatively slow, potentially resulting in prolonged exposure to the peak temperature range for δ-phase precipitation, thereby leading to increased δ-phase formation.

### 3.2. HE Susceptibility

**Figure 3:**
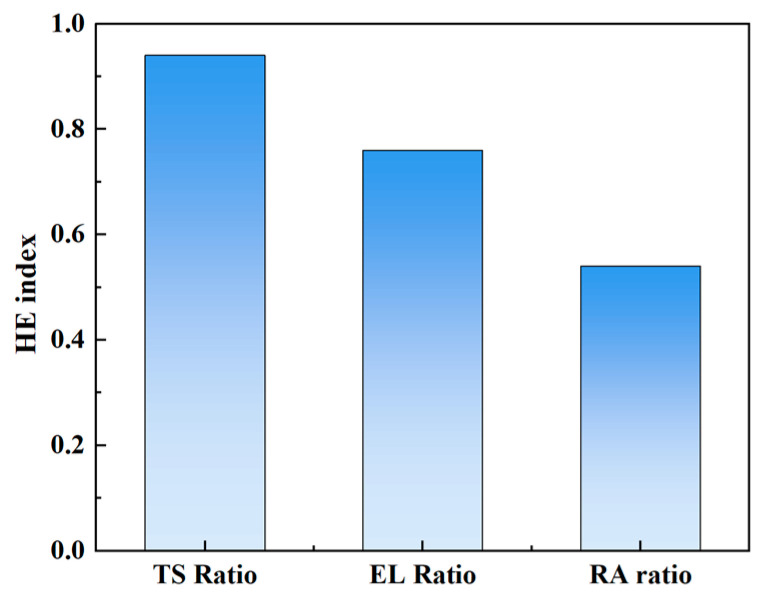
Hydrogen embrittlement susceptibility of GH625.

Table 2 presents the tensile results of the H-charged and H-uncharged specimens. The ratio of the mechanical properties between the H-charged and H-uncharged samples was used as an index to evaluate hydrogen embrittlement. The specific results are shown in Figure 3. Hydrogen has a relatively small effect on tensile strength (TS), with the tensile strength ratio being approximately 0.94. In contrast, the ductility loss is more pronounced, with the elongation (EL) ratio being about 0.76 and the reduction of area (RA) ratio being about 0.54. The loss in RA is significantly more sensitive to hydrogen.

### 3.3. Fractographic Observation

**Figure 4:**
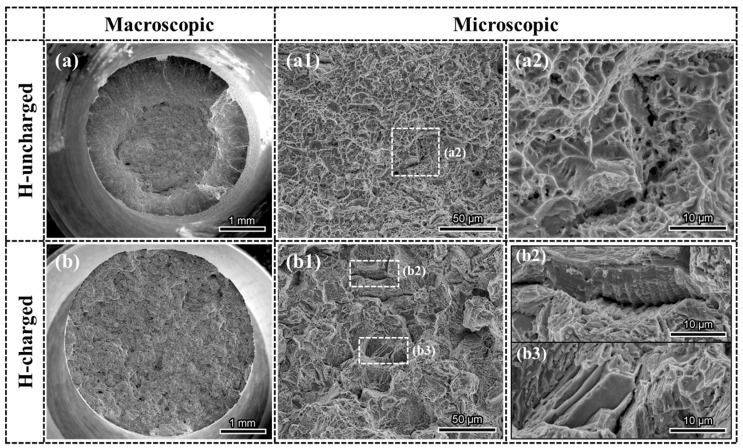
SEM images of fracture surface morphologies: (**a**,**a1**,**a2**) H-uncharged specimen; (**b**,**b1**–**b3**) H-charged specimen.

To understand the hydrogen embrittlement behavior of GH625, it is essential to examine the tensile fracture. The SEM morphology of the tensile fracture is presented in Figure 4. The H-uncharged sample exhibits a microvoid coalescence-induced ductile fracture. The macroscopic fracture is clearly divided into two regions: the fibrous area and the shear lip area. A large number of fine dimples are observed in the fibrous area, with fine particles present at the bottom of the dimples. Additionally, a few secondary cracks are found, as shown in Figure 4(a2). These cracks are connected by fine dimples, displaying a ductile fracture characteristic. The fracture morphology of the material changes significantly after hydrogen charging. The macroscopic fracture shows no distinct partition. From the magnified image in Figure 4(b2), numerous intergranular secondary cracks are visible, with a quasi-cleavage morphology evident on the grain surfaces. These features indicate that the material transitions to a mixed fracture mode of ductility and brittleness after hydrogen charging. Figure 4(b2) shows an enlarged image of a secondary crack at a grain boundary. Obvious slip traces and numerous second-phase particles can be observed within the crack. Additionally, some smooth facets with slip traces are also present. According to the findings of Ogawa et al. [20] on Inconel 718, these smooth facets may result from the separation of slip planes (SPs) or TBs. The slip traces are formed due to the interaction between DSBs or with twin boundaries. This type of area constitutes a relatively small proportion.

### 3.4. Deformation Microstructure

**Figure 5:**
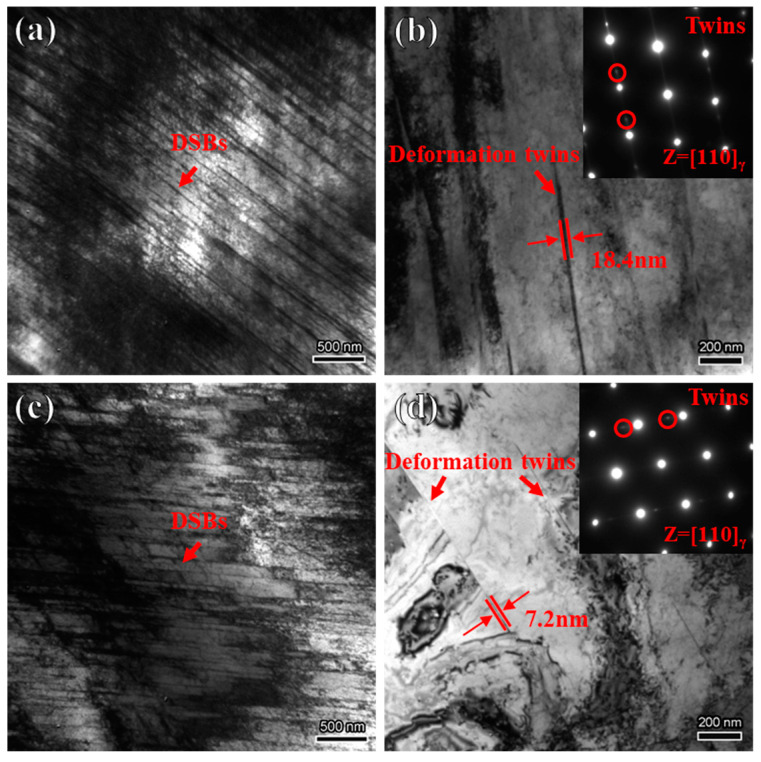
TEM results of fractured samples: (**a**,**b**) H-uncharged; (**c**,**d**) H-charged.

The deformation microstructure formed during tensile testing is essential for understanding the fracture mechanism; therefore, the microstructure of the specimens after the tensile test was characterized in detail. After tensile deformation, the microstructure of the specimen is primarily composed of parallel DSBs, with a small number of deformation twins also present. As shown in Figure 5b,d, the thickness of the deformation twins in the H-charged specimen decreases, with the average thickness reducing from 18.4 nm to 7.2 nm, consistent with the results reported by Liu et al. [27].

### 3.5. Observation of Hydrogen-Induced Cracks

**Figure 6:**
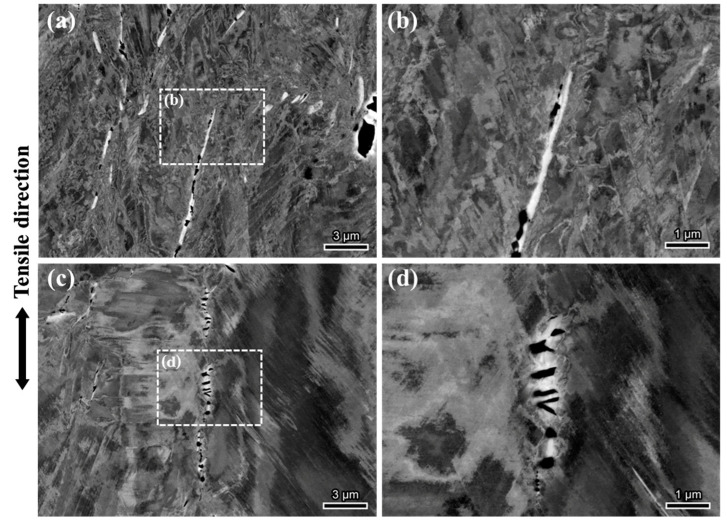
ECCI image near the fracture of the tensile sample: (**a**,**b**) H-uncharged specimen; (**c**,**d**) H-charged specimen.

The ECCI image of the area near the fracture surface is shown in Figure 6. Obvious plane slip bands were observed in the sample without hydrogen, and the microvoids mainly initiated near the δ phase at the grain boundary (as shown in Figure 6a). Due to the typically high interfacial energy between the precipitates and the matrix, deformation between the precipitates and the matrix is not well coordinated during plastic deformation, which easily results in localized stress concentrations and subsequently leads to the formation of microvoids. As the number of voids near the precipitates increases, some of the voids may coalesce to form cracks (as shown in Figure 4(a2)). After hydrogen charging, voids still tend to initiate at grain boundaries. Additionally, the δ phase at grain boundaries is found to be prone to fracture along the tensile direction (TD), a phenomenon rarely observed in H-uncharged specimens. This suggests that hydrogen promotes the cracking of the δ phase. It is speculated that δ-phase cracking is closely associated with the formation of intergranular cracks.

**Figure 7:**
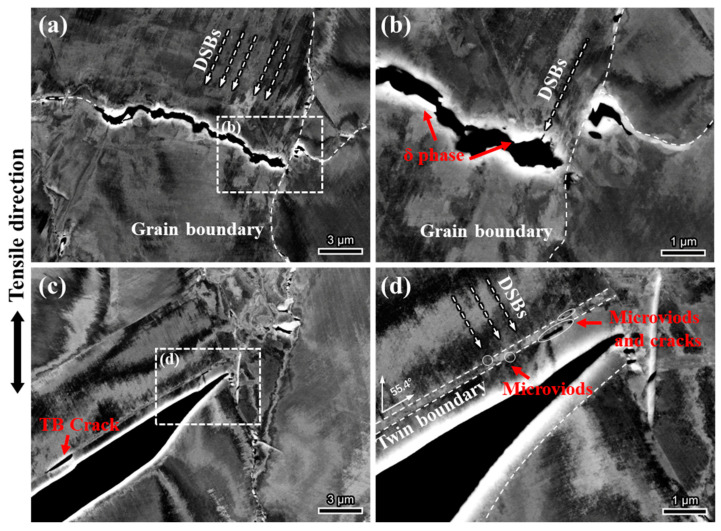
ECCI image of secondary cracks in H-charged specimen: (**a**,**b**) random grain boundary crack; (**c**,**d**) twin boundary crack.

To investigate the hydrogen embrittlement mechanism of the H-charged sample, the cracks in the H-charged sample were further observed, as shown in Figure 7. Two main types of cracks were identified: one type of crack formed along the random grain boundaries where the δ phase was present, and the other type of crack formed along the twinning boundaries. Figure 7b shows an intergranular crack with a rough crack interface. The enlarged image reveals an evident δ phase within the crack. The interaction between the dislocation slip band and the δ phase is observed, leading to stress concentration and subsequent cracking of the δ phase under the influence of hydrogen. The formation of intergranular cracks results from the coalescence of δ-phase voids at grain boundaries, as shown in Figure 6c. Grain boundaries perpendicular to the tensile direction are more prone to intergranular cracking. Although needle-shaped δ phases were found in some twin boundaries, it was observed that twin boundaries could still serve as crack nucleation sites even in the absence of the δ phase. Figure 7c shows a larger twin boundary crack with a relatively straight crack interface. DSBs perpendicular to the twin boundary are clearly visible within the twin, further indicating the planar slip characteristics of the material. Small voids and cracks are also observed along the adjacent twin boundaries.

## 4. Discussion

### 4.1. Hydrogen-Induced Random GB Cracking

Random grain boundaries distributed particles of the δ phase are the primary sites for hydrogen-induced crack initiation in this study. In polycrystalline metals, the accumulation of dislocations near grain boundaries, combined with elastic constraints in the surrounding areas, typically leads to localized deformation distinct from that within the grain interior. This strain accommodation process necessitates the activation of enough slip systems to ensure compatibility between adjacent grains. However, in precipitation-strengthened alloys or solid-solution-strengthened alloys with high alloy content, the shearing of coherent precipitates by moving dislocations and the presence of short-range order lead to pronounced planar dislocation slip in the alloy [31,32,33]. Planar slip limits the ability of dislocations to cross-slip, and reduces their three-dimensional mobility. This limitation hinders effective strain coordination near grain boundaries, making these regions more susceptible to fracture [20]. Grain boundaries also serve as hydrogen trapping sites [34]. Mobile dislocations within the grains transport hydrogen to these boundaries [35]. Additionally, random grain boundaries act as short-range diffusion pathways, enhancing the absorption and movement of hydrogen in polycrystalline materials [36]. The segregation of hydrogen at grain boundaries reduces the critical strain for material failure, increasing the likelihood of intergranular cracking.

**Figure 8:**
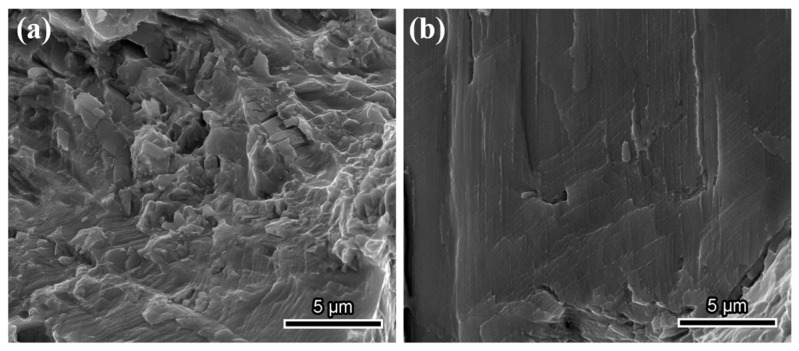
SEM microscopic images of different types of fractures: (**a**) random grain boundary fracture; (**b**) twin boundary fracture.

In this study, a large amount of particles of δ phase were found at the grain boundaries. The δ phase is believed to play a key role in the hydrogen-induced cracking process of superalloys [37,38]. The relationship between hydrogen distribution and the δ phase, however, remains controversial. Some studies [39] suggest that the hydrogen solubility in the δ phase is lower than in the matrix, leading to hydrogen accumulation primarily at the δ/γ interface. This accumulation weakens interatomic bonding, resulting in hydrogen embrittlement via the HEDE mechanism, characterized by a relatively smooth brittle zone on the fracture surface without obvious signs of deformation. In contrast, other studies [40] suggest that the δ phase has significantly higher hydrogen solubility relative to the matrix. In this case, there is no pronounced hydrogen accumulation at the δ/γ interface, but the high hydrogen content within the δ phase also results in a higher hydrogen content near the interface. In this study, no exposed δ phase was observed in the fracture surfaces of the H-uncharged specimen. However, as shown in Figure 8, large pieces of δ phases were visible in the fractures of the H-charged specimen, suggesting that hydrogen reduces the bonding strength at the δ/γ interface, facilitating the initiation of interface cracks. Additionally, cracked δ phases were observed in both Figure 6 and Figure 8. This observation supports the hypothesis that hydrogen has higher solubility in the δ phase. Dissolved hydrogen increases the brittleness of the δ phase, making it more susceptible to cracking during deformation. Strain concentration at the grain boundaries further exacerbates this phenomenon, and the cracking of the δ phase contributes to the propagation of intergranular cracks.

In precipitation-strengthened alloys, the formation of the δ phase can lead to precipitation-free zones (PFZs) at grain boundaries [41], which increase strain concentration near these boundaries. Although GH625 is primarily a solid solution-strengthened superalloy, it can also precipitate the γ’’ phase during aging [2]. In this study, the alloy was aged at 680 °C; however, due to the short aging time, no γ’’-phase precipitation was observed.

### 4.2. Hydrogen-Induced TB Cracking

In this study, twin boundaries were identified as secondary initiation sites for hydrogen-induced cracks in GH625. Twin boundaries are completely coherent interfaces and are generally considered more resistant to hydrogen embrittlement due to their lower interfacial energy and hydrogen solubility [42]. However, in some superalloys, twin boundaries have been observed to serve as preferential initiation sites for hydrogen-induced cracks [20,43]. Similar to random grain boundaries, twin boundaries present a strong barrier to dislocation motion. Some studies suggest that crack initiation at twin boundaries is linked to the accumulation of dislocations at these sites [43]. As discussed earlier, GH625 exhibits characteristics of planar dislocation slip. According to the HELP mechanism, atomic hydrogen inhibits the cross-slip of dislocations [44], further promoting planar slip and causing strain localization. The dilatational stress field near dislocation slip bands and changes in the local shear modulus lead to hydrogen concentration near dislocations, forming a hydrogen cloud [45]. This hydrogen cloud reduces interactions between dislocations and obstacles, allowing dislocations to move under lower stress [46]. Consequently, dislocations and hydrogen accumulate near twin boundaries, resulting in localized strain enhancement. Additionally, in face-centered cubic structures, both twin planes and slip planes are {111} planes, which allows dislocations to slip along twin planes. This promotes the formation of dislocation substructures at the interface, making twin boundaries potential sites for crack nucleation [47]. Figure 8b shows slip traces on the twin boundary fracture, indicating clear interaction between the TB and DSBs. As shown in Figure 7, under uniaxial stress, twin boundary cracks generally form at an angle of approximately 60° to the tensile direction, whereas random grain boundary cracks tend to form closer to 90°. This suggests that, in addition to normal stress, twin boundaries are also significantly influenced by shear stress, which promotes boundary sliding and facilitates crack initiation.

In addition to annealing twins, deformation twins are also present in GH625. Deformation twins typically form in materials with low stacking fault energy (SFE). The SFE of Inconel 625 is relatively low (~30 mJ/m^2^) [48], resulting in a deformation mechanism primarily involving dislocation slip and deformation twinning. In the research of Liu et al. [27], the deformation mechanism of Inconel 625 alloy under slow strain rate tension is mainly a dislocation slip, while at conventional rates, deformation twins form, producing the TWIP effect. Hydrogen weakens the TWIP effect by refining deformation twins and also promotes the formation of twin bundles. These twin bundles, composed of multiple small twins, lead to increased strain concentration, making them more prone to cracking. The number of deformation twins observed in this study was relatively small, likely because deformation twins typically form during the later stages of deformation [49]. However, the δ phase at the grain boundaries reduces the alloy’s ductility, leading to fracture before a substantial number of deformation twins can form. Consequently, deformation twins have little impact on the hydrogen embrittlement of this alloy.

### 4.3. Summary of Hydrogen-Induced Cracking Mechanism in GH625

**Figure 9:**
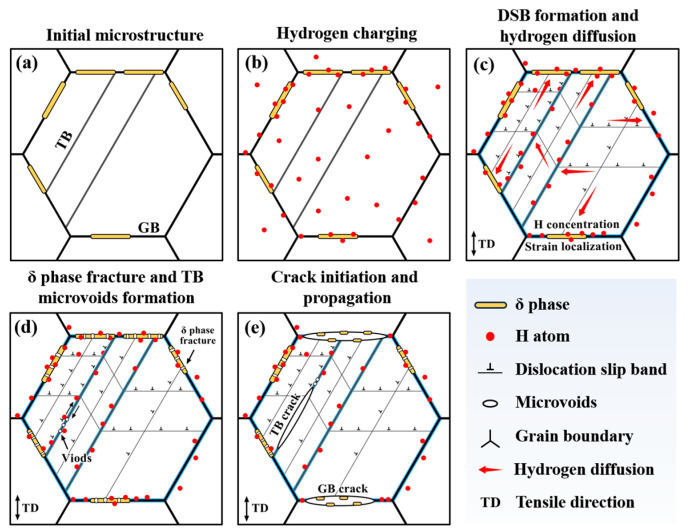
Initiation and propagation mechanism of hydrogen-induced cracks in GH625 alloy.

In summary, the specific deformation mechanism of GH625 under thermal hydrogen-charging conditions is illustrated in Figure 9. After heat treatment, a significant amount of δ-phase precipitates at the grain boundaries (as shown in Figure 9a). Following hydrogen charging, the alloy reaches a saturated hydrogen state. Due to the high hydrogen trap capacity and solubility of the δ phase, its internal hydrogen content becomes higher than that of the matrix (as shown in Figure 9b). Upon the onset of plastic deformation, numerous DSBs form within the alloy. According to the HELP mechanism, hydrogen promotes localized plastic deformation and accelerates dislocation movement, leading to dislocation accumulation at various interfaces, including random grain boundaries, twin boundaries, and δ/γ interfaces. This accumulation significantly increases local stress at the interfaces. Simultaneously, hydrogen within the grains segregates to the interfaces alongside dislocation movement (as shown in Figure 9c). Due to the higher hydrogen content in the δ phase, its critical fracture stress is reduced, leading to fracture under applied stress. According to the HEDE mechanism, the elevated hydrogen content at the interface weakens the bonding strength between interfaces. Under the combined effects of local stress concentration and hydrogen accumulation, twin boundaries in favorable orientations slide, resulting in the formation of micropore defects (as shown in Figure 9d). As plastic deformation intensifies, the δ/γ interface fractures due to hydrogen accumulation and local dislocation pile-up, propagating through voids created by earlier δ-phase fractures to form random grain boundary cracks. Microvoids along the twin boundary coalesce into microcracks, which propagate along the boundary to form twin boundary fractures, ultimately forming a fracture mechanism characterized by dominant random grain boundary cracking, with twin boundary cracking playing a secondary role (as shown in Figure 9e).

## 5. Conclusions

In this study, the effect of hydrogen on the tensile properties of GH625 was investigated through tensile testing following thermal hydrogen charging. The mechanism of hydrogen-induced crack initiation and propagation was analyzed using microscopic characterization, leading to the following conclusions:

1. After pre-hydrogen charging, the tensile ductility of GH625 is significantly reduced, with an HE index of 0.76 for elongation and 0.54 for area reduction. The primary deformation mechanism is planar dislocation slip, with a small presence of deformation twins.
2. Random grain boundary fracture is the primary hydrogen-induced fracture mode in the alloy. Hydrogen-induced δ-phase fracture and debonding at the δ/γ interface are the primary causes of intergranular fracture. Local strain concentration, resulting from hydrogen-enhanced planar dislocation slip, promotes δ-phase cracking and crack propagation along grain boundaries.
3. Hydrogen-induced twin boundary cracking serves as an auxiliary fracture mechanism. This is primarily due to hydrogen-induced local stress concentration promoting twin boundary sliding and hydrogen segregation reducing twin boundary cohesion strength.

## Figures and Tables

**Table 1:** Chemical composition of the GH625.

C	Si	Mn	Cr	Mo	Nb	Ti	Al	Fe	Ni
0.011	0.04	0.005	21.79	8.92	3.68	0.27	0.16	0.18	Bal.

**Table 2:** Tensile properties of GH625.

	Tensile Strength/MPa	Elongation/%	Reduction of Area/%
H-uncharged	1077 ± 5.43	37 ± 1.22	52 ± 1.38
H-charged	1010 ± 6.11	28 ± 1.15	28 ± 2.51

## Data Availability

The original contributions presented in this study are included in the article. Further inquiries can be directed to the corresponding authors.
